# Pneumonia as a predictor of diabetes mellitus and coronary heart disease: a national cohort study

**DOI:** 10.1186/s41479-026-00199-x

**Published:** 2026-05-05

**Authors:** Filip Jansåker, Xinjun Li, Kristina Sundquist, Henning Stenberg

**Affiliations:** 1 Center for Primary Health Care Research, Department of Clinical Sciences, Malmö, Lund University, Malmö. Jan Waldenströms gata 35, Skåne University Hospital, Malmö, 20502 Sweden; 2 University Clinic Primary Care, Skåne University Hospital, Region Skåne, Malmö, Sweden; 3 Department of Clinical Microbiology, Rigshospitalet, Blegdamsvej 9, Copenhagen, DK- 2100 Denmark; 4 Center for Community-Based Healthcare Research and Education (CoHRE), Department of Functional Pathology, School of Medicine, Shimane University, 1060 Nishikawatsu-cho, Matsue, Shimane, 690-8504 Japan

**Keywords:** Epidemiology, Pneumonia, Diabetes mellitus, Coronary heart disease, Infections

## Abstract

**Background:**

Pneumonia is a common infection that leads to frequent hospitalizations and primary healthcare visits. Previous smaller studies have indicated high prevalence of undiagnosed diabetes mellitus (DM) and increased subsequent risk of coronary heart disease (CHD) among patients with pneumonia. However, previous studies have not used nationwide data that include diagnoses from primary healthcare settings, where most pneumonias are treated. The aim of this study was to examine whether pneumonia is associated with subsequent DM and CHD.

**Methods:**

This was an open nationwide cohort study of adults 35–75 years of age in Sweden 2007–2018, including national registers and population-based primary healthcare data. The outcomes were DM and CHD, and individuals with outcomes diagnosed before the index date (including 2002–2005) were excluded. The index date was set as the first pneumonia diagnosis or the first healthcare contact (in those without pneumonia) during the study period. Cox regression models were used to estimate hazard ratios (HR) and 95% confidence intervals (95% CI) while adjusting for potential confounders.

**Results:**

A total of 4,580,606 individuals without previously diagnosed DM and 4,661,052 individuals without previously diagnosed CHD were included; of these, 348,024 individuals were diagnosed with DM and 295,592 with CHD during follow-up, respectively. Pneumonia preceded DM in 104,598 (30.1%) and CHD in 94,087 (31.8%) individuals. Compared with no diagnosis, pneumonia was associated with an age-adjusted HR of 1.12 (95% CI 1.11–1.13) for DM and 1.18 (95% CI 1.17–1.19) for CHD. In the full model, pneumonia was associated with a HR of 1.11 (95% CI 1.10–1.12) for both outcomes. Several complementary analyses were conducted, showing significant associations across most age-groups, in both sexes, across different follow-up periods (e.g. <1 year and ≥ 10 years), and in patients diagnosed with pneumonia in primary healthcare settings.

**Conclusions:**

This nationwide study found that pneumonia is associated with subsequent DM and CHD. The findings indicate that pneumonia has a potential role as a clinical predictor of DM or CHD, including in primary healthcare settings, which warrants further clinical studies.

**Supplementary Information:**

The online version contains supplementary material available at 10.1186/s41479-026-00199-x.

## Background

Early diagnosis and treatment of diabetes mellitus (DM) are essential for preventing cardiovascular complications, such as coronary heart disease (CHD) [1]. However, for the most common form of DM (i.e. type 2 diabetes), the time from onset of DM until diagnosis is long (5–6 years), likely due to unspecific early symptoms [2–4].

DM is a risk factor for infections, including pneumonia [5]. Comprehensive studies on the associations between DM and pneumonia are, however, scarce [6]. A recent review of observational studies reported increased occurrence of pneumonia among patients with DM, but the included studies lacked large population-based primary healthcare data and were judged to be at serious risk of bias [7]. To what extent pneumonia precedes DM diagnoses is also largely unknown. In a recent cohort study of patients admitted for pneumonia, undiagnosed DM was prevalent in 5% and undiagnosed prediabetes in 37.5%, but this study did not include any controls (e.g. patients admitted for other reasons than pneumonia) [8]. Several studies have found that the risk of CHD increases following respiratory tract infections [9–14]. However, studies including population-based data from primary healthcare (where most patients with pneumonia are treated) examining the associations between pneumonia and subsequent DM and CHD are lacking. Apart from estimating these associations, such studies may identify whether pneumonia could be a useful clinical predictor of DM or CHD.

By using almost complete nationwide primary healthcare data and comprehensive national population registers, we aimed to examine associations between pneumonia and subsequent DM as well as pneumonia and subsequent CHD in a nationwide cohort. We also aimed to examine the risks of DM and CHD following pneumonia diagnosis in different age-groups, settings, and follow-up periods.

## Methods

### Design and Setting

A nationwide open cohort study that included over 4.6 million individuals aged 35–75 years with a registered healthcare contact in Sweden during the study period (2007–2018) was conducted. The index date was set as the first pneumonia diagnosis or the first registered healthcare contact (for those without pneumonia) during the study period. The study used data from national registers and almost complete nationwide primary healthcare data. Linkages between the registers were performed using the national unique civic registration number [15].

### Study population

Figure S1 shows a flowchart of the study population selection. First, we identified individuals aged 35–75 years with at least one healthcare contact during the study period (2007–2018). Individuals aged < 35 or > 75 years at the index date were not included. We also excluded individuals with preexisting outcomes diagnosed before index date, including a period of five years (2002–2006) prior to the start of the study period. The 10th revision of the International Classification of Diseases (ICD-10) code “E11” (type 2 diabetes) were used to exclude patients with prior DM and ICD-10 codes “I20–I25” (coronary heart diseases) were used for the exclusion of those with previously diagnosed CHD. To ensure that we did not include any patients with prior DM diagnosis, we also excluded those with a redeemed prescription of an antidiabetic drug—Anatomic Therapeutic Chemical (ATC) classification system code “A10” (all medications for DM, including insulin)—between July 2005 and the index date.

### Predictor variable

The main predictor was defined as the first episode of pneumonia during the study period, based on the 10th revision of the International Classification of Diseases (ICD-10) codes (‘J10.0’, ‘J11.0’, ‘J12.0–J18.1’, ‘J18.3–J18.9’).

#### Outcome variables

The two main outcomes in the study were: (1) DM, based on redeemed antidiabetic prescriptions (ATC code “A10”, including insulin) or ICD-10 code “E11” during the follow-up period; and (2) CHD, measured as ICD-10 codes “I20–I25”. Other types of diabetes (e.g. gestational diabetes, ICD-10 code “O24”) were not assessed.

####  Covariates

*Sociodemographic factors* were collected at the index date. Sex was included as a categorical variable: male or female. *Age* was categorized into four groups: 35–44, 45–54, 55–64, and 65–75 years of age in the descriptive table but defined as a continuous variable (increasing by each year) in the statistical analysis. *Family history* of DM or CHD (any parent or sibling) was defined as yes or no. *Country of origin* was categorized into born in Sweden or not (born outside of Sweden). *Region of residence* was categorized into two groups: residing in the three largest Swedish cities or not. *Socioeconomic status* was defined by individual education and family income. *Educational level* was based on the duration of school years attended. The following categories were used: < 12 years or ≥ 12 years. *Family income* was categorized into two groups based on a weighted average income in each family: low (lowest two income quartiles of the study population) and high (the highest two quartiles). *Medical comorbidities* or risk factors for DM and CHD [16] were assessed during the study period, i.e. chronic obstructive pulmonary disease (COPD, ICD-10 codes: J40–J49); obesity (ICD-10 E65–E68); alcoholism (ICD-10 F10, K70); hypertension (ICD-10 I10–I19), and heart failure (ICD-10 I50). *Body-mass-index* (BMI), a known risk factor for DM and CHD [17], was included in sensitivity analyses for parous women [18] and men who went through mandatory military enlistment [19].

###  Data sources

The study population was identified in the National Patient Register (NPR) and primary healthcare data. The predictor (pneumonia) and outcomes (DM and CHD) were also identified using data derived from primary healthcare settings (available for 1997–2018) and the National Patient Register (NPR), encompassing data from both inpatient (1964–2018) and outpatient care (2001–2018). The Swedish Prescribed Drug Register was used to collect data on redeemed prescriptions of antidiabetic drugs (available from July 2005 to 2018), used as a proxy for DM, to ensure that all patients with DM were identified. The Medical Birth Register (MBR, 1973–2018) and the Swedish Military Conscription Register (1969–2018) contains data on BMI for parous women and men who went through military enlistment, respectively. Population based BMI data was not available in any of the other data sources that were used in this study. The Total Population Register (TPR, 1968–2018) and the Multi-Generation Register (MGR, 1932–2018) were used to collect data on death, emigration, immigration, sociodemographic variables and family relations. The TPR and the MGR are managed by the Swedish governmental authority, *Statistics Sweden* (SCB). NPR, MBR, and Swedish Prescribed Drug Register are nationwide registers managed by the National Board of Health and Welfare (in Swedish: *Socialstyrelsen*). These registers include the entire Swedish population with little missing data. Furthermore, diagnoses in the NPR are > 99% complete and have been reported to have a high positive predictive value across several evaluated diagnoses [20, 21], including for DM (~ 99% in one study [22]). The nationwide primary healthcare data includes data from 20 out of 21 administrative regions in Sweden during the study period. The coverage of these data varied by time and region based on when patient records became digitalized; the coverage was around 72% of the Swedish population in 2015 and around 90% at the end of the study [23]. Individuals with missing values (range 0.2–3.9%) on the sociodemographic variables were not excluded: i.e. missing values on education level (3.9%), family income (0.8%), region of residence (0.8%), and country of origin (0.2%) were instead included in the categories low education level, low family income, large cities, and born in Sweden, respectively.

### Statistical analysis

Descriptive characteristics of the study population, number of cases, and incidence proportions per 100 persons of each outcome during the follow-up were calculated for the main predictor variable (pneumonia) and the covariates. The incidence rates per 1000 person-years for individuals with and without pneumonia, were plotted for each outcome by age. To test for associations between the predictor variable and the outcomes, Cox regression models were used to estimate hazard ratios (HR) and 95% confidence intervals (95% CI). The study period started on January 1, 2007, and data on the predictor variable (pneumonia) were collected for individuals aged 35–75 years during the entire study period. The index date was set as the date of the first pneumonia diagnosis or the first healthcare contact (any causes, for those without pneumonia) during the study period. Follow-up proceeded from the index date until outcome (DM or CHD), death, emigration, or end of the study period (December 31, 2018), whichever came first. A crude model and three adjusted models were used in the main analysis for each outcome: Model 1, age-adjusted model; Model 2, adjusted for age, sex, individual sociodemographic factors and family history; and Model 3, a full model that included all covariates in the adjustments. Family history differed in the two analyses (i.e. family history of DM in the DM analysis and family history of CHD in the CHD analysis). Hypertension and heart failure were only included in the adjustment in the CHD models. We also conducted several complementary analyses: We estimated DM and CHD risks following pneumonia stratified by settings and ages (groups) at index date. We also conducted an additional analysis stratified by follow-up periods. For this analysis, we used an indirect standardization methods, enabling us to estimate the incidence of DM and CHD during different follow-up periods (< 1, 1–4, 5–9, and ≥ 10 years) after pneumonia diagnosis, controlled for all covariates. The results from this analysis are shown as standardized incidence ratios (SIRs) for DM and CHD) in those with a preceding pneumonia compared to those without. A sensitivity analysis, including BMI, was also conducted for DM. We also explored graded associations between pneumonia and the outcomes (including p-values for trends), stratifying by the number of pneumonia events after the index date (first pneumonia diagnosis) during the study period. This was the only analysis in which temporality was not preserved, i.e. some of the repeated pneumonia events could have occurred after the outcome. A two-tailed p-value of < 0.05 was considered statistically significant. The proportional hazard assumption was tested by plotting the incidence rates over time (Figure S2-S3), and by calculating Schoenfeld (partial) residuals—the assumption was fulfilled. All statistical analyses were performed using SAS 9.4 (SAS Institute Inc.; Cary, NC, USA).

## Results

Table 1 shows the study population, consisting of 4,580,606 individuals without a previous diagnosis of DM and 4,661,052 individuals without a previous CHD diagnosis. Among these, the incidence proportion of DM (348,024 cases) was 7.60% (95% CI 7.57–7.62) and 6.34% (95% CI 6.32–6.36) for CHD (295,592 cases) during follow-up, respectively. The number of individuals with a pneumonia diagnosis before a DM diagnosis was 104,598, i.e. 7.06% of those with pneumonia and 30.1% of those diagnosed with DM during follow-up. For CHD, the corresponding numbers were 94,087, i.e. 6.22% of those with pneumonia and 31.8% of those diagnosed with CHD during follow-up. Detailed characteristics are presented in Supplementary Tables S1-S4.

**Table 1:** Characteristics of study population, number of cases, and incidence proportions of diabetes mellitus and coronary heart disease during follow-up, 2007–2018 (Sweden)

	Population		Cases		Incidence proportions		
	No.	%	No.	%	%	95% CI	
Diabetes mellitus							
Total^1^	4,580,606	100.0	348,024	100.0	7.60	7.57	7.62
Pneumonia							
Non	3,099,885	67.7	243,426	69.9	7.85	7.82	7.88
Diagnosis^3^	1,480,721	32.3	104,598	30.1	7.06	7.02	7.11
Sex							
Males	2,290,556	50.0	200,581	57.6	8.76	8.72	8.80
Females	2,290,050	50.0	147,443	42.4	6.44	6.41	6.47
Age (years)							
35–44	1,267,553	27.7	41,806	12.0	3.30	3.27	3.33
45–54	1,173,042	25.6	74,575	21.4	6.36	6.31	6.40
55–64	1,122,174	24.5	112,375	32.3	10.01	9.96	10.07
65–75	1,017,837	22.2	119,268	34.3	11.72	11.65	11.78
Coronary heart disease							
Total^2^	4,661,052	100.0	295,592	100.0	6.34	6.32	6.36
Pneumonia							
Non	3,148,359	67.5	201,505	68.2	6.40	6.37	6.43
Diagnosis^3^	1,512,693	32.5	94,087	31.8	6.22	6.18	6.26
Sex							
Males	2,326,185	49.9	183,124	62.0	7.87	7.84	7.91
Females	2,334,867	50.1	112,468	38.0	4.82	4.79	4.85
Age (years)							
35–44	1,283,541	27.5	16,033	5.4	1.25	1.23	1.27
45–54	1,194,934	25.6	44,877	15.2	3.76	3.72	3.79
55–64	1,148,842	24.6	91,410	30.9	7.96	7.91	8.01
65–75	1,033,735	22.2	143,272	48.5	13.86	13.79	13.93

CI = confidence interval. ^1^ Individuals with a diagnosis of diabetes mellitus any time within five years or a redeemed prescription of antidiabetic drug within two years before the study period were excluded. ^2^Individuals with a diagnosis of coronary heart disease at any time within five years before the study period were excluded. ^3^Not including pneumonia events occurring after diabetes mellitus and coronary heart disease during the study period. Detailed characteristics are included in Supplementary Tables S1-S4

Pneumonia was significantly associated with subsequent DM and subsequent CHD in the crude models: HR 1.15 (95% CI 1.14–1.16) for DM and HR 1.22 (95% CI 1.21–1.23) for CHD. Table 2 (for DM) and Table 3 (for CHD) show that the associations decreased slightly in the age-adjusted models but remained significant. In the full model, the HR was 1.11 (95% CI 1.10–1.12) for both outcomes in individuals with a preceding pneumonia compared to those without—i.e. 1.106 (95% CI 1.097–1.115) for DM and 1.113 (95% CI 1.104–1.123) for CHD (three decimals are not included in the tables).

**Table 2:** Association between pneumonia and subsequent diabetes mellitus in Swedish patients aged 35–75 years, 2007–2018 (Sweden)

	Model 1			Model 2			Model 3		
Covariates	HR	95% CI		HR	95% CI		HR	95% CI	
Pneumonia (ref. no preceding diagnosis)	1.12	1.11	1.13	1.16	1.15	1.16	1.11	1.10	1.12
Age (per one-year increase)	1.04	1.04	1.04	1.05	1.04	1.05	1.04	1.04	1.04
Male sex (ref. females)				1.61	1.60	1.62	1.59	1.58	1.60
Educational level (ref. ≥ 12 years)				1.34	1.33	1.35	1.30	1.29	1.31
Family income (ref. high)				1.20	1.19	1.21	1.18	1.18	1.19
Region of residence (ref. large cities)				1.16	1.15	1.17	1.15	1.14	1.16
Country of origin (ref. born in Sweden)				2.10	2.09	2.12	2.09	2.07	2.10
Family history^1^ (ref. no)				1.84	1.83	1.85	1.81	1.80	1.82
COPD (ref. no diagnosis)							0.95	0.93	0.96
Alcoholism (ref. no diagnosis)							1.12	1.10	1.14
Obesity (ref. no diagnosis)							2.86	2.81	2.90
Hypertension (ref. no diagnosis)							1.73	1.71	1.74
Heart failure (ref. no diagnosis)							1.33	1.31	1.35

HR = hazard ratio, CI = confidence interval, COPD = chronic obstructive pulmonary disease. Crude model: HR 1.15 (95% CI 1.14–1.16); Model 1: Age adjusted model; Model 2: Including age, sex, individual sociodemographic factors and family history in the adjustments. Model 3: Full model. ^1^Family historyof diabetes mellitus. Age was defined at baseline and analyzed as a continuous variable (per one-year increase). Data on diagnoses of pneumonia and diabetes mellitus were collected from primary healthcare data and the National Patient Register. Data on redeemed prescriptions of an antidiabetic drug (proxy for diabetes mellitus diagnosis) were collected from the National Prescribed Drug Register

**Fig. 1:**
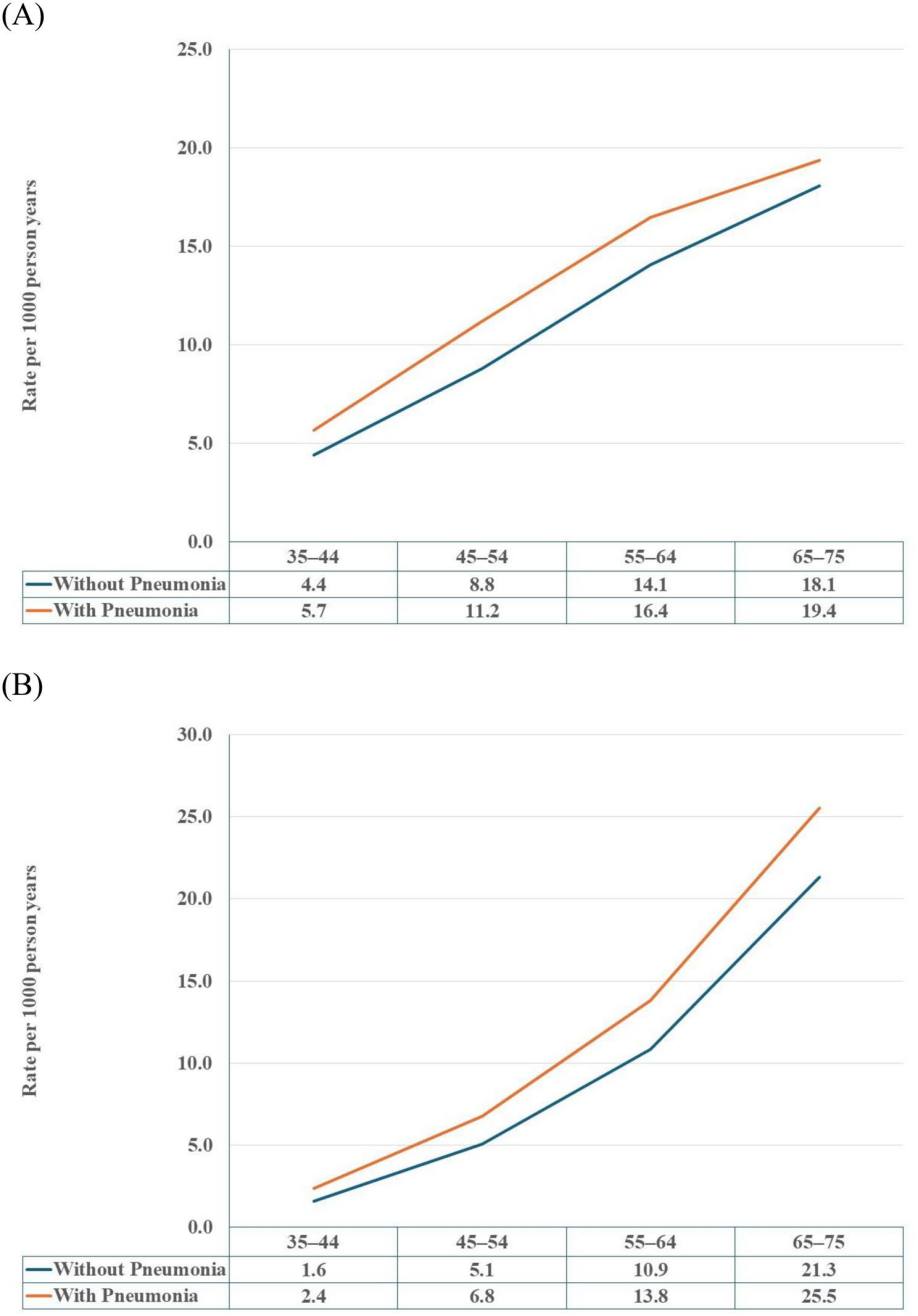
Incidence rate (per 1000 person-years) of diabetes mellitus (**A**) and coronary heart disease (**B**) in patients with and without a preceding pneumonia by age. The first row under the x-axis denotes age-groups. The second and third row denote the incidence rate per 1000 person years for diabetes mellitus (**A**) and coronary heart disease (**B**) for individuals without and with pneumonia during the study period, respectively

Figure 1 shows that the incidence rates of DM and CHD were higher in individuals with a preceding pneumonia than in those without across all age-groups during follow-up. Table 4 shows that pneumonia was associated with a HR of 1.20 (95% CI 1.18–1.23) for DM and 1.45 (95% CI 1.40–1.50) for CHD in individuals aged 35–44 years at the index date. The associations appeared to attenuate with advanced age for both outcomes, with a HR of 1.04 (95% CI 1.03–1.06) for CHD and no association observed for DM in individuals aged 65–75 years.

**Table 3:** Association between pneumonia and subsequent coronary heart disease in Swedish patients aged 35–75 years, 2007–2018 (Sweden)

	Model 1			Model 2			Model 3		
Covariates	HR	95% CI		HR	95% CI		HR	95% CI	
Pneumonia (ref. no preceding diagnosis)	1.18	1.17	1.19	1.25	1.24	1.26	1.11	1.10	1.12
Age (per one-year increase)	1.08	1.08	1.08	1.08	1.08	1.08	1.06	1.06	1.06
Male sex (ref. females)				2.01	1.99	2.02	1.89	1.87	1.90
Educational level (ref. ≥ 12 years)				1.28	1.27	1.29	1.21	1.20	1.22
Family income (ref. high)				1.18	1.17	1.19	1.13	1.12	1.14
Region of residence (ref. large cities)				1.25	1.24	1.26	1.23	1.22	1.24
Country of origin (ref. born in Sweden)				1.59	1.57	1.60	1.58	1.56	1.59
Family history^1^ (ref. no)				1.40	1.39	1.41	1.39	1.38	1.40
COPD (ref. no diagnosis)							1.22	1.21	1.24
Alcoholism (ref. no diagnosis)							0.97	0.95	0.99
Obesity (ref. no diagnosis)							1.25	1.22	1.27
Hypertension (ref. no diagnosis)							2.65	2.62	2.67
Heart failure (ref. no diagnosis)							2.85	2.82	2.88

HR = hazard ratio, CI = confidence interval, COPD = chronic obstructive pulmonary disease. Crude model: HR 1.22 (95% CI 1.21–1.23); Model 1: Age adjusted model; Model 2: Including age, sex, individual sociodemographic factors and family history in the adjustments. Model 3: Full model. ^1^Family history ^2^of diabetes mellitus. Age was defined at baseline and analyzed as a continuous variable (per one-year increase). Data on diagnoses of pneumonia and coronary heart disease were collected from primary healthcare data and the National Patient Register

**Table 4:** Associations between pneumonia and subsequent diabetes mellitus and coronary heart disease, by different age groups, 2007–2018 (Sweden)

Age at index date (years)	Diabetes mellitus					Coronary heart disease				
	Cases	HR^1^	95% CI			Cases	HR^1^	95% CI		
35–44	41,806	1.20	1.18	1.23		16,033	1.45	1.40	1.50	
45–54	74,575	1.16	1.14	1.18		44,877	1.16	1.14	1.19	
55–64	112,375	1.09	1.07	1.10		91,410	1.09	1.07	1.11	
65–75	119,268	1.00	0.99	1.02		143,272	1.04	1.03	1.06	

HR = hazard ratio, CI = confidence interval. ^1^ Full model, adjusted for sociodemographic factors, family history, and comorbidities—alcoholism, chronic obstructive pulmonary disease, and obesity, as well as hypertension and heart failure (for the coronary heart disease analysis)

In the sensitivity analysis including BMI (Supplementary Table S5), a graded association between BMI and DM was observed in both sexes. Pneumonia remained associated with subsequent DM in both men and women when including BMI in the adjustments: the HR for DM was 1.36 (95% CI 1.32–1.40) in women and 1.10 (95% CI 1.08–1.12) in men with a preceding pneumonia compared to women and men without, respectively.

In the analysis stratified by the number of pneumonia events (Supplementary Table S6), a graded association was observed between pneumonia and the outcomes. Individuals with two events had a HR of 1.07 (95% CI 1.05–1.08) for DM and 1.07 (95% CI 1.05–1.09) for CHD, compared with those without pneumonia, and those with five or more events had the highest HR of DM and CHD: 1.24 (95% CI 1.23–1.26) and 1.26 (95% CI 1.24–1.27), respectively.

In the analysis stratified by setting of pneumonia diagnosis (Supplementary Table S7), pneumonia diagnosed in primary healthcare settings was associated with a HR of 1.14 (95% CI 1.13–1.15) for subsequent DM and 1.12 (95% CI 1.11–1.14) for subsequent CHD compared with no pneumonia. In inpatient settings, pneumonia was associated with a HR of 1.22 (95% CI 1.19–1.24) for DM and 1.29 (95% CI 1.27–1.32) for CHD, whereas the association between pneumonia and CHD was weaker and no association was observed for DM in outpatient specialist care settings.

In the analysis stratified by follow-up time (Supplementary Table S8), pneumonia was significantly associated with subsequent DM and CHD across all follow-up periods (< 1, 1–4, 5–9, and ≥ 10 years), with the exception for CHD within 1–4 years of follow-up. The SIRs appeared highest within < 1 year and ≥ 10 years following pneumonia. Within < 1 year of follow-up, the SIR was 1.66 (95% CI 1.63–1.68) for DM (20,293 observations) and 1.90 (95% CI 1.88–1.93) for CHD (22,254 observations). After ≥ 10 years of follow-up, the SIR was 1.81 (95% CI 1.76–1.87) for DM (4516 observations) and 1.33 (95% CI 1.29–1.38) for CHD (3479 observations). The overall SIRs for DM (1.28, 95% CI 1.27–1.29) and CHD (1.19, 95% CI 1.19–1.20) following pneumonia were comparable to the main findings (Tables 2 and 3).

## Discussion

### Summary

In this nationwide follow-up study, pneumonia was significantly associated with both subsequent DM and CHD. To the best of our knowledge, this is the first study to examine the associations between pneumonia and subsequent DM and CHD in a nationwide setting including data from national healthcare registers and primary healthcare settings. The findings suggest a potential use of pneumonia as a clinical predictor of DM and CHD, meriting further examination in clinical studies.

### Comparison with existing literature

The increased risk of pneumonia in patients with DM has several explanations, such as an impaired immune system and higher glucose levels, facilitating infections [4, 5, 7, 24, 25]. Together with the diagnostic delay (5–6 years) in DM [2–4] this may explain the higher risk of DM following pneumonia diagnosis. A previous study showed a 5% prevalence of undiagnosed DM and 37.5% prevalence of undiagnosed prediabetes in patients diagnosed with pneumonia in hospital settings (cumulatively 42.5%) [8]. In our study, 7.06% of those with pneumonia were subsequently diagnosed with DM. Although we did not have access to blood tests to determine the prevalence of undiagnosed prediabetes or DM at the time of pneumonia diagnosis, it is plausible that these prevalences may be lower in individuals treated for pneumonia in primary healthcare; however, this warrants further studies. Nevertheless, DM risk was elevated following pneumonia diagnosed in both primary healthcare and hospital settings, albeit with indications of a stronger association in inpatients settings, where pneumonia severity is likely greater. The association between pneumonia and CHD appears to be more complex and could be bidirectional. Like DM, CHD seems to be a risk factor for pneumonia [26] and earlier studies have also indicated that pneumonia might increase the risk of a cardiovascular event [9–14], possibly ≥ 10 years following the pneumonia [27]. We also observed elevated CHD risk both short-term and long-term after pneumonia, including pneumonia diagnosed in primary healthcare. Although our observational study cannot establish any causality, several potential mechanisms exist by which pneumonia could contribute to CHD [28] and evidence of casual relationships to DM has also recently emerged [29]. The findings of graded associations together with the elevated risks that persisted for several years after pneumonia and after adjustments for potentially shared risk factors (e.g. sociodemographic factors, comorbidities, and BMI) [16, 17, 27, 30] may contribute evidence to the literature of a potential causal association between pneumonia and CHD—possibly also DM—which merits further studies.

### Limitations and strengths

The main limitations are that we lack data on the severity of disease and clinical presentation, laboratory and radiological findings. We could therefore not validate the diagnoses or directly address different levels of severity of pneumonia. First, potential misclassification of pneumonia is probably most substantial in primary healthcare settings where the access to diagnostic procedures (e.g. imaging) is more limited than in hospital settings. Second, although DM and CHD risks were significantly elevated following pneumonia diagnosed in inpatient (presumed higher severity) as well as in primary healthcare setting (presumed lower severity), the risks appeared higher in inpatient settings. This suggests that the associations could depend on pneumonia severity, which warrant future examinations in clinical studies. Third, although the prevalence of undiagnosed DM and prediabetes in patients with validated pneumonia diagnosis has been examined in hospital settings [8], further studies with access to these types of data from primary healthcare are needed. Moreover, data on potential confounders (e.g. smoking), was also lacking. Lastly, certain antidiabetic drugs could have been prescribed for other conditions during the study period. However, we are only aware of one such indication that could cause a potential misclassification bias during the study period, i.e. metformin for polycystic ovary syndrome. Given that only 0.075% of all women with redeemed prescription of metformin also had this diagnosis registered during the study period, this would likely not affect the main findings. Our study also has several strengths that balances its limitations. The major strengths of this study were that we had access to almost complete nationwide primary healthcare data (from 20 of 21 regions in Sweden) and national registers of high completeness, including almost all individuals diagnosed with pneumonia, CHD and DM during the study period. The inclusion of diagnoses registered in both primary healthcare, specialist clinics, and hospitals ensures that the results are relevant for the entire population and for clinicians in all types of healthcare facilities. The expected findings, i.e. that age, sex, BMI, family history, and certain individual sociodemographic factors were associated with DM and CHD, were consistent with previous studies [16, 31], confirming that the data sources were reliable.

### Clinical implications and further research

The associations found in this nationwide study suggest that pneumonia could be a useful independent clinical predictor of subsequent DM and CHD, including in primary healthcare settings. The findings also indicate that pneumonia may not be useful as a predictor of DM in patients ≥ 65 years—possibly due to the high background risk and/or high prevalence of already diagnosed DM in this age-group [16]. For clinicians, our findings support maintaining a high level of suspicion for undiagnosed DM and imminent CHD events in most patients ≥ 35 years with symptoms of—or recent diagnosis of—pneumonia. Although clinical studies are needed before a general recommendation can be made, testing for undiagnosed DM in patients with pneumonia [8] may also be considered in those managed in primary healthcare settings. In addition, screening and optimizing treatment for DM as well as other CHD risk factors (e.g. hypertension and hyperlipidemia) may help mitigate the elevated CHD risk in these patients, although this remains to be directly studied. From a public health perspective, the long-term elevation in DM and CHD risks, persisting more than a decade after pneumonia—alongside plausible causal mechanisms [28, 29] and vaccines [32, 33] for pneumonia causing pathogens—suggest that pneumonia may be a potentially modifiable risk factor for these conditions. This is further supported by a recent meta-analysis showing decreased cardiovascular disease risk in pneumococcal [32] and influenza [33] vaccine recipients, meriting to examine potential similar effects for DM as well as to evaluate whether these vaccines could be useful, cost-effective strategies for DM and CHD prevention. Lastly, our study is based on register data from Sweden—a high-income country with universal healthcare coverage and a relatively low prevalence of undiagnosed DM compared to many other countries [34, 35]. Therefore, our findings may hold particular importance but require validation in other populations and settings, such as in low-income countries where undiagnosed DM is more prevalent [35].

## Conclusions

Pneumonia was strongly associated with both subsequent DM and CHD in a nationwide setting including data from national healthcare registers and primary healthcare settings. These findings indicates that pneumonia may be a useful independent clinical predictor for DM and CHD in Sweden, where the prevalence of undiagnosed DM is considered low, which warrants to be examined in clinical studies. Further studies should also be done in certain risk populations or in settings where the prevalence of undiagnosed DM is higher.

## Supplementary Information

Below is the link to the electronic supplementary material.

Supplementary Material 1

Supplementary Material 2

## Data Availability

This study made use of several national registers and, owing to legal concerns, data cannot be made openly available. Further information regarding the health registries is available from the Swedish National Board of Health and Welfare: https://www.socialstyrelsen.se/en/statistics-and-data/registers/ and Statistics Sweden: https://www.scb.se/en/services/ordering-data-and-statistics/.

## Abbreviations

ATC: Anatomic Therapeutic Chemical classification system
BMI: Body Mass Index
CHD: Coronary Heart Disease
CI: Confidence Interval
COPD: Chronic Obstructive Pulmonary Disease
DM: Diabetes Mellitus
HR: Hazard Ratio
ICD: International Classification of Diseases
MBR: Medical Birth Register
MGR: Multi-Generation Register
NPR: National Patient Register
SCB: Statistics Sweden
TPR: Total Population Register
