# Supplementary material for: Pneumonia as a predictor of diabetes mellitus and coronary heart disease: a national cohort study
Source: Pneumonia (Nathan). 2026 May 5;18:10. doi: 10.1186/s41479-026-00199-x (PMC13141523; doi:10.1186/s41479-026-00199-x)
Supplement: Supplementary file 1 — Supplementary Material 1 [file 41479_2026_199_MOESM1_ESM.docx]

| **Table S1.** Characteristics of study population, number of cases, and incidence proportions of diabetes mellitus during follow-up, 2007–2018 (Sweden) | | | | | | | | | |
| --- | --- | --- | --- | --- | --- | --- | --- | --- | --- |
|  | **Population** | |  | **Cases** | |  | **Incidence proportions** | | |
|  | No. | % |  | No. | % |  | % | 95% CI | |
| **Education** |  |  |  |  |  |  |  |  |  |
| ≤ 12 | 2,368,127 | 51.7 |  | 223,770 | 64.3 |  | 9.45 | 9.41 | 9.49 |
| > 12 | 2,212,479 | 48.3 |  | 124,254 | 35.7 |  | 5.62 | 5.58 | 5.65 |
| **Family income** |  |  |  |  |  |  |  |  |  |
| Lowest two quartiles | 2,287,851 | 49.9 |  | 178,918 | 51.4 |  | 7.82 | 7.78 | 7.86 |
| Highest two quartiles | 2,292,755 | 50.1 |  | 169,106 | 48.6 |  | 7.38 | 7.34 | 7.41 |
| **Region of residence** |  |  |  |  |  |  |  |  |  |
| Large cities | 2,379,812 | 52.0 |  | 162,542 | 46.7 |  | 6.83 | 6.80 | 6.86 |
| Outside of large cities | 2,200,794 | 48.0 |  | 185,482 | 53.3 |  | 8.43 | 8.39 | 8.47 |
| **Country of origin** |  |  |  |  |  |  |  |  |  |
| Born in Sweden | 3,756,416 | 82.0 |  | 271,366 | 78.0 |  | 7.22 | 7.20 | 7.25 |
| Born outside of Sweden | 824,190 | 18.0 |  | 76,658 | 22.0 |  | 9.30 | 9.24 | 9.37 |
| **Family history^1^** |  |  |  |  |  |  |  |  |  |
| Non | 3,444,914 | 75.2 |  | 232,210 | 66.7 |  | 6.74 | 6.71 | 6.77 |
| Yes | 1,135,692 | 24.8 |  | 115,814 | 33.3 |  | 10.20 | 10.14 | 10.26 |
| **COPD** |  |  |  |  |  |  |  |  |  |
| Non | 4,358,497 | 95.2 |  | 323,826 | 93.0 |  | 7.43 | 7.40 | 7.46 |
| Diagnosis | 222,109 | 4.8 |  | 24,198 | 7.0 |  | 10.89 | 10.76 | 11.03 |
| **Alcoholism** |  |  |  |  |  |  |  |  |  |
| Non | 4,456,372 | 97.3 |  | 335,872 | 96.5 |  | 7.54 | 7.51 | 7.56 |
| Diagnosis | 124,234 | 2.7 |  | 12,152 | 3.5 |  | 9.78 | 9.61 | 9.96 |
| **Obesity** |  |  |  |  |  |  |  |  |  |
| Non | 4,488,030 | 98.0 |  | 329,046 | 94.5 |  | 7.33 | 7.31 | 7.36 |
| Diagnosis | 92,576 | 2.0 |  | 18,978 | 5.5 |  | 20.50 | 20.21 | 20.79 |
| **Hypertension** |  |  |  |  |  |  |  |  |  |
| Non | 3,878,368 | 84.7 |  | 230,258 | 66.2 |  | 5.94 | 5.91 | 5.96 |
| Diagnosis | 702,238 | 15.3 |  | 117,766 | 33.8 |  | 16.77 | 16.67 | 16.87 |
| **Heart** **failure** |  |  |  |  |  |  |  |  |  |
| Non | 4,458,113 | 97.3 |  | 324,477 | 93.2 |  | 7.28 | 7.25 | 7.30 |
| Diagnosis | 122,493 | 2.7 |  | 23,547 | 6.8 |  | 19.22 | 18.98 | 19.47 |
| **Total** | 4,580,606 | 100.0 |  | 348,024 | 100.0 |  | 7.60 | 7.57 | 7.62 |
| CI = confidence interval, COPD = chronic obstructive pulmonary disease. ^1^ Family history of diabetes mellitus. All individuals with a diagnosis of diabetes mellitus any time within five years or a redeemed prescription of an antidiabetic drug within two years before the study period were excluded. | | | | | | | | | |

| **Table S2.** Characteristics of study population and number of cases of diabetes mellitus during follow-up, 2007–2018 (Sweden) | | | | | | | | |
| --- | --- | --- | --- | --- | --- | --- | --- | --- |
|  | **DM cases without preceding pneumonia** | |  | **DM cases following pneumonia** | |  | **All DM cases** | |
|  | No. | % |  | No. | % |  | No. | % |
| **Sex** |  |  |  |  |  |  |  |  |
| Males | 147,425 | 60.6 |  | 53,156 | 50.8 |  | 200,581 | 57.6 |
| Females | 96,001 | 39.4 |  | 51,442 | 49.2 |  | 147,443 | 42.4 |
| **Age** |  |  |  |  |  |  |  |  |
| 35–44 | 29,269 | 12.0 |  | 12,537 | 12.0 |  | 41,806 | 12.0 |
| 45–54 | 52,050 | 21.4 |  | 22,525 | 21.5 |  | 74,575 | 21.4 |
| 55–64 | 77,593 | 31.9 |  | 34,782 | 33.3 |  | 112,375 | 32.3 |
| 65–75 | 84,514 | 34.7 |  | 34,754 | 33.2 |  | 119,268 | 34.3 |
| **Education** |  |  |  |  |  |  |  |  |
| ≤ 12 | 156,636 | 64.3 |  | 67,134 | 64.2 |  | 223,770 | 64.3 |
| > 12 | 86,790 | 35.7 |  | 37,464 | 35.8 |  | 124,254 | 35.7 |
| **Family income** |  |  |  |  |  |  |  |  |
| Lowest two quartiles | 124,652 | 51.2 |  | 54,266 | 51.9 |  | 178,918 | 51.4 |
| Highest two quartiles | 118,774 | 48.8 |  | 50,332 | 48.1 |  | 169,106 | 48.6 |
| **Region of residence** |  |  |  |  |  |  |  |  |
| Large cities | 107,523 | 44.2 |  | 55,019 | 52.6 |  | 162,542 | 46.7 |
| Outside of large cities | 135,903 | 55.8 |  | 49,579 | 47.4 |  | 185,482 | 53.3 |
| **Country of origin** |  |  |  |  |  |  |  |  |
| Born in Sweden | 188,546 | 77.5 |  | 82,820 | 79.2 |  | 271,366 | 78.0 |
| Born outside of Sweden | 54,880 | 22.5 |  | 21,778 | 20.8 |  | 76,658 | 22.0 |
| **Family history^1^** |  |  |  |  |  |  |  |  |
| Non | 164,084 | 67.4 |  | 68,126 | 65.1 |  | 232,210 | 66.7 |
| Yes | 79,342 | 32.6 |  | 36,472 | 34.9 |  | 115,814 | 33.3 |
| **COPD** |  |  |  |  |  |  |  |  |
| Non | 241,548 | 99.2 |  | 82,278 | 78.7 |  | 323,826 | 93.0 |
| Diagnosis | 1878 | 0.8 |  | 22,320 | 21.3 |  | 24,198 | 7.0 |
| **Alcoholism** |  |  |  |  |  |  |  |  |
| Non | 235,938 | 96.9 |  | 99,934 | 95.5 |  | 335,872 | 96.5 |
| Diagnosis | 7488 | 3.1 |  | 4664 | 4.5 |  | 12,152 | 3.5 |
| **Obesity** |  |  |  |  |  |  |  |  |
| Non | 233,032 | 95.7 |  | 96,014 | 91.8 |  | 329,046 | 94.5 |
| Diagnosis | 10394 | 4.3 |  | 8584 | 8.2 |  | 18,978 | 5.5 |
| **Hypertension** |  |  |  |  |  |  |  |  |
| Non | 163,813 | 67.3 |  | 66,445 | 63.5 |  | 230,258 | 66.2 |
| Diagnosis | 79,613 | 32.7 |  | 38,153 | 36.5 |  | 117,766 | 33.8 |
| **Heart** **failure** |  |  |  |  |  |  |  |  |
| Non | 229,074 | 94.1 |  | 95,403 | 91.2 |  | 324,477 | 93.2 |
| Diagnosis | 14,352 | 5.9 |  | 9195 | 8.8 |  | 23,547 | 6.8 |
| **Total** | 243,426 | 100.0 |  | 104,598 | 100.0 |  | 348,024 | 100.0 |
| DM = Diabetes mellitus, CI = confidence interval, COPD = chronic obstructive pulmonary disease. ^1^ Family history of diabetes mellitus. All individuals with a diagnosis of diabetes mellitus any time within five years or a redeemed prescription of an antidiabetic drug within two years before the study period were excluded. | | | | | | | | |

| **Table S3.** Characteristics of study population and number of cases, and incidence proportions of coronary heart disease during follow-up (2007–2018) | | | | | | | | | |
| --- | --- | --- | --- | --- | --- | --- | --- | --- | --- |
|  | **Population** | |  | **Cases** | |  | **Incidence proportions** | | |
|  | No. | % |  | No. | % |  | % | 95% CI | |
| **Education** |  |  |  |  |  |  |  |  |  |
| ≤ 12 | 2,420,680 | 51.9 |  | 193,307 | 65.4 |  | 7.99 | 7.95 | 8.02 |
| > 12 | 2,240,372 | 48.1 |  | 102,285 | 34.6 |  | 4.57 | 4.54 | 4.59 |
| **Family income** |  |  |  |  |  |  |  |  |  |
| Lowest two quartiles | 2,330,593 | 50.0 |  | 143,276 | 48.5 |  | 6.15 | 6.12 | 6.18 |
| Highest two quartiles | 2,330,459 | 50.0 |  | 152,316 | 51.5 |  | 6.54 | 6.50 | 6.57 |
| **Region of residence** |  |  |  |  |  |  |  |  |  |
| Large cities | 2,419,562 | 51.9 |  | 129,527 | 43.8 |  | 5.35 | 5.32 | 5.38 |
| Outside of large cities | 2,241,490 | 48.1 |  | 166,065 | 56.2 |  | 7.41 | 7.37 | 7.44 |
| **Country of origin** |  |  |  |  |  |  |  |  |  |
| Born in Sweden | 3,820,074 | 82.0 |  | 245,662 | 83.1 |  | 6.43 | 6.41 | 6.46 |
| Born outside of Sweden | 840,978 | 18.0 |  | 49,930 | 16.9 |  | 5.94 | 5.89 | 5.99 |
| **Family history^1^** |  |  |  |  |  |  |  |  |  |
| Non | 3,016,786 | 64.7 |  | 172,238 | 58.3 |  | 5.71 | 5.68 | 5.74 |
| Yes | 1,644,266 | 35.3 |  | 123,354 | 41.7 |  | 7.50 | 7.46 | 7.54 |
| **COPD** |  |  |  |  |  |  |  |  |  |
| Non | 4,439,333 | 95.2 |  | 265,059 | 89.7 |  | 5.97 | 5.95 | 5.99 |
| Diagnosis | 221,719 | 4.8 |  | 30,533 | 10.3 |  | 13.77 | 13.62 | 13.93 |
| **Alcoholism** |  |  |  |  |  |  |  |  |  |
| Non | 4,533,224 | 97.3 |  | 286,160 | 96.8 |  | 6.31 | 6.29 | 6.34 |
| Diagnosis | 127,828 | 2.7 |  | 9432 | 3.2 |  | 7.38 | 7.23 | 7.53 |
| **Obesity** |  |  |  |  |  |  |  |  |  |
| Non | 4,556,539 | 97.8 |  | 286,209 | 96.8 |  | 6.28 | 6.26 | 6.30 |
| Diagnosis | 104,513 | 2.2 |  | 9383 | 3.2 |  | 8.98 | 8.80 | 9.16 |
| **Hypertension** |  |  |  |  |  |  |  |  |  |
| Non | 3,934,145 | 84.4 |  | 151,161 | 51.1 |  | 3.84 | 3.82 | 3.86 |
| Diagnosis | 726,907 | 15.6 |  | 144,431 | 48.9 |  | 19.87 | 19.77 | 19.97 |
| **Heart** **failure** |  |  |  |  |  |  |  |  |  |
| Non | 4,541,340 | 97.4 |  | 249,619 | 84.4 |  | 5.50 | 5.48 | 5.52 |
| Diagnosis | 119,712 | 2.6 |  | 45,973 | 15.6 |  | 38.40 | 38.05 | 38.76 |
| **Total** | 4,661,052 | 100.0 |  | 295,592 | 100.0 |  | 6.34 | 6.32 | 6.36 |
| CI = confidence interval, COPD = chronic obstructive pulmonary disease. ^1^ Family history of coronary heart disease. All individuals with a diagnosis of coronary heart disease at any time within five years before the study period were excluded. | | | | | | | | | |

| **Table S4.** Characteristics of study population and number of cases of coronary heart disease during follow-up (2007–2018) | | | | | | | | |
| --- | --- | --- | --- | --- | --- | --- | --- | --- |
|  | **CHD cases without preceding pneumonia** | |  | **CHD cases following pneumonia** | |  | **All CHD cases** | |
|  | No. | % |  | No. | % |  | No. | % |
| **Sex** |  |  |  |  |  |  |  |  |
| Males | 131,482 | 65.2 |  | 51,642 | 54.9 |  | 183,124 | 62.0 |
| Females | 70,023 | 34.8 |  | 42,445 | 45.1 |  | 112,468 | 38.0 |
| **Age** |  |  |  |  |  |  |  |  |
| 35–44 | 10,683 | 5.3 |  | 5350 | 5.7 |  | 16,033 | 5.4 |
| 45–54 | 30,777 | 15.3 |  | 14,100 | 15.0 |  | 44,877 | 15.2 |
| 55–64 | 61,375 | 30.5 |  | 30,035 | 31.9 |  | 91,410 | 30.9 |
| 65–75 | 98,670 | 49.0 |  | 44,602 | 47.4 |  | 143,272 | 48.5 |
| **Education** |  |  |  |  |  |  |  |  |
| ≤ 12 | 130,950 | 65.0 |  | 62,357 | 66.3 |  | 193,307 | 65.4 |
| > 12 | 70,555 | 35.0 |  | 31,730 | 33.7 |  | 102,285 | 34.6 |
| **Family income** |  |  |  |  |  |  |  |  |
| Lowest two quartiles | 95,820 | 47.6 |  | 47,456 | 50.4 |  | 143,276 | 48.5 |
| Highest two quartiles | 105,685 | 52.4 |  | 46,631 | 49.6 |  | 152,316 | 51.5 |
| **Region of residence** |  |  |  |  |  |  |  |  |
| Large cities | 82,761 | 41.1 |  | 46,766 | 49.7 |  | 129,527 | 43.8 |
| Outside of large cities | 118,744 | 58.9 |  | 47,321 | 50.3 |  | 166,065 | 56.2 |
| **Country of origin** |  |  |  |  |  |  |  |  |
| Born in Sweden | 167,687 | 83.2 |  | 77,975 | 82.9 |  | 245,662 | 83.1 |
| Born outside of Sweden | 33,818 | 16.8 |  | 16,112 | 17.1 |  | 49,930 | 16.9 |
| **Family history^1^** |  |  |  |  |  |  |  |  |
| Non | 118,786 | 58.9 |  | 53,452 | 56.8 |  | 172,238 | 58.3 |
| Yes | 82,719 | 41.1 |  | 40,635 | 43.2 |  | 123,354 | 41.7 |
| **COPD** |  |  |  |  |  |  |  |  |
| Non | 197,931 | 98.2 |  | 67,128 | 71.3 |  | 265,059 | 89.7 |
| Diagnosis | 3574 | 1.8 |  | 26,959 | 28.7 |  | 30,533 | 10.3 |
| **Alcoholism** |  |  |  |  |  |  |  |  |
| Non | 196,320 | 97.4 |  | 89,840 | 95.5 |  | 286,160 | 96.8 |
| Diagnosis | 5185 | 2.6 |  | 4247 | 4.5 |  | 9432 | 3.2 |
| **Obesity** |  |  |  |  |  |  |  |  |
| Non | 196,705 | 97.6 |  | 89,504 | 95.1 |  | 286,209 | 96.8 |
| Diagnosis | 4800 | 2.4 |  | 4583 | 4.9 |  | 9383 | 3.2 |
| **Hypertension** |  |  |  |  |  |  |  |  |
| Non | 102,931 | 51.1 |  | 48,230 | 51.3 |  | 151,161 | 51.1 |
| Diagnosis | 98,574 | 48.9 |  | 45,857 | 48.7 |  | 144,431 | 48.9 |
| **Heart** **failure** |  |  |  |  |  |  |  |  |
| Non | 172,463 | 85.6 |  | 77,156 | 82.0 |  | 249,619 | 84.4 |
| Diagnosis | 29,042 | 14.4 |  | 16,931 | 18.0 |  | 45,973 | 15.6 |
| **Total** | 201,505 | 100.0 |  | 94,087 | 100.0 |  | 295,592 | 100.0 |
| CHD = coronary heart disease, CI = confidence interval, COPD = chronic obstructive pulmonary disease. ^1^ Family history of coronary heart disease. All individuals with a diagnosis of coronary heart disease at any time within five year before the study period were excluded. | | | | | | | | |

| **Table S5.** Sensitivity analysis on the associations between pneumonia and subsequent diabetes mellitus in women and men aged 35–75 years, including body mass index in the adjustments, 2007–2018 (Sweden) | | | | | | | |
| --- | --- | --- | --- | --- | --- | --- | --- |
|  | **Women** (n=674,057) | | |  | **Men** (n=1,147,012) | | |
| Covariates | HR^1^ | 95% CI | |  | HR^1^ | 95% CI | |
| **Pneumonia** (ref. no preceding diagnosis) | 1.36 | 1.32 | 1.40 |  | 1.10 | 1.08 | 1.12 |
| **Age** (per one-year increase) | 1.08 | 1.07 | 1.08 |  | 1.09 | 1.09 | 1.09 |
| **Educational level** (ref. ≥ 12 years) | 1.38 | 1.34 | 1.41 |  | 1.33 | 1.31 | 1.35 |
| **Family income** (ref. high) | 1.26 | 1.22 | 1.29 |  | 1.13 | 1.11 | 1.15 |
| **Region of residence** (ref. large cities) | 1.39 | 1.36 | 1.43 |  | 1.19 | 1.17 | 1.20 |
| **Country of origin** (ref. born in Sweden) | 2.52 | 2.44 | 2.61 |  | 1.40 | 1.35 | 1.46 |
| **Family history^2^** (ref. no) | 2.10 | 2.04 | 2.16 |  | 2.04 | 2.01 | 2.07 |
| **COPD** (ref. no diagnosis) | 1.22 | 1.16 | 1.29 |  | 1.06 | 1.01 | 1.10 |
| **Alcoholism** (ref. no diagnosis) | 1.38 | 1.27 | 1.50 |  | 1.38 | 1.33 | 1.42 |
| **Obesity** (ref. no diagnosis) | 2.06 | 1.97 | 2.15 |  | 2.86 | 2.77 | 2.96 |
| **BMI^3^** (ref. 18.5–24.9) |  |  |  |  |  |  |  |
| <18.5 | 0.83 | 0.77 | 0.90 |  | 0.87 | 0.85 | 0.90 |
| 25.0–29.9 | 2.28 | 2.21 | 2.36 |  | 2.14 | 2.09 | 2.19 |
| ≥ 30.0 | 4.05 | 3.89 | 4.23 |  | 3.02 | 2.90 | 3.14 |
| BMI = body mass index, HR = hazard ratio, CI = confidence interval, COPD = chronic obstructive pulmonary disease. ^1^ Full model, including all covariates and BMI in the adjustments. ^2^ Family history of diabetes mellitus. ^3^ BMI for men was collected at enlistment from the Military Conscription Register and BMI for women was collected at pregnancy from the Swedish Medical Birth Register. All individuals with a diagnosis of diabetes mellitus at any time within five years or redeemed prescriptions of an antidiabetic drug within two years before the study period were excluded. | | | | | | | |

| **Table S6.** Associations between pneumonia and diabetes mellitus, and pneumonia and coronary heart disease, by the number of infections, 2007–2018 (Sweden) | | | | | | | | | | | | |
| --- | --- | --- | --- | --- | --- | --- | --- | --- | --- | --- | --- | --- |
|  | **Diabetes mellitus** | | | | |  | **Coronary heart disease** | | | | | |
|  | (4,580,606 adults aged 35–75 years) | | | | |  | (4,661,052 adults aged 35–75 years) | | | | | |
| Number of pneumonias (ref. no) | Cases | HR^1^ | 95% CI | | Trend test p-value | | Cases | HR^1^ | 95% CI | | Trend test p-value |  |
| One | 25,833 | 0.99 | 0.98 | 1.01 | <.001 |  | 20,875 | 0.99 | 0.97 | 1.00 | <.001 |  |
| Two | 16,747 | 1.07 | 1.05 | 1.08 |  |  | 14,026 | 1.07 | 1.05 | 1.09 |  |  |
| Three | 10,144 | 1.13 | 1.11 | 1.15 |  |  | 8607 | 1.13 | 1.10 | 1.15 |  |  |
| Four | 7542 | 1.13 | 1.10 | 1.15 |  |  | 6736 | 1.15 | 1.12 | 1.18 |  |  |
| Five or more | 44,332 | 1.24 | 1.23 | 1.26 |  |  | 43,843 | 1.26 | 1.24 | 1.27 |  |  |
| HR = hazard ratio, CI = confidence interval. ^1^ Full model, adjusted for sociodemographic factors (age, sex, educational level, family income, region of residence, and country of origin), family history, and comorbidities—alcoholism, chronic obstructive pulmonary disease, and obesity, as well as hypertension and heart failure (for the coronary heart disease analysis). | | | | | | | | | | | | |

| **Table S7.** The association between pneumonia and subsequent diabetes mellitus and coronary heart disease by setting (2007–2018) | | | | | | | | | |
| --- | --- | --- | --- | --- | --- | --- | --- | --- | --- |
| Setting of pneumonia diagnosis  (ref. no preceding pneumonia diagnosis) | **Diabetes mellitus^1^** | | | |  | **Coronary heart disease^2^** | | | |
|  | Cases | HR^1^ | 95% CI | |  | Cases | HR^1^ | 95% CI | |
| **Inpatient specialist care** | 11,934 | 1.22 | 1.19 | 1.24 |  | 15,452 | 1.29 | 1.27 | 1.32 |
| **Outpatient specialist care** | 20,043 | 0.99 | 0.98 | 1.01 |  | 18,795 | 1.05 | 1.03 | 1.07 |
| **Primary healthcare** | 72,621 | 1.14 | 1.13 | 1.15 |  | 59,840 | 1.12 | 1.11 | 1.14 |
| ^1^ Full model, adjusted for sociodemographic factors (age, sex, educational level, family income, region of residence, and country of origin), family history, and comorbidities (alcoholism, chronic obstructive pulmonary disease, and obesity, as well as hypertension and heart failure [coronary heart disease only]). ^1^ All individuals with a diagnosis of diabetes mellitus at any time within five years or redeemed prescriptions of an antidiabetic drug within two years before the study period were excluded. ^2^ All individuals with a diagnosis of coronary heart disease at any time within five years before the study period were excluded. | | | | | | | | | |

| **Table S8.** Standardized incidence ratios for diabetes mellitus and coronary heart disease during different follow-up periods after pneumonia diagnosis, 2007–2018 (Sweden) | | | | | | | | | | | |
| --- | --- | --- | --- | --- | --- | --- | --- | --- | --- | --- | --- |
|  | **Diabetes mellitus** | | | | |  | **Coronary heart disease** | | | | |
| Follow-up time (years) | O | E | SIR | 95% CI | |  | O | E | SIR | 95% CI | |
| <1 | 20,293 | 12,246.44 | **1.66** | **1.63** | **1.68** |  | 22,254 | 11,710.98 | **1.90** | **1.88** | **1.93** |
| 1–4 | 47,953 | 44,295.6 | **1.08** | **1.07** | **1.09** |  | 42,235 | 42,777.84 | 0.99 | 0.98 | 1.00 |
| 5–9 | 31,836 | 22,557.78 | **1.41** | **1.40** | **1.43** |  | 26,119 | 21,719.94 | **1.20** | **1.19** | **1.22** |
| ≥10 | 4516 | 2493.07 | **1.81** | **1.76** | **1.87** |  | 3479 | 2606.95 | **1.33** | **1.29** | **1.38** |
| All | 104,598 | 81,592.88 | **1.28** | **1.27** | **1.29** |  | 94,087 | 78,815.72 | **1.19** | **1.19** | **1.20** |
| O=Observed; E=Expected; SIR=Standardized incidence ratio; CI=Confidence interval Bold type: 95% confidence interval does not include 1.00. | | | | | | | | | | | |
