# Supplementary material for: Pneumonia as a predictor of diabetes mellitus and coronary heart disease: a national cohort study
Source: Pneumonia (Nathan). 2026 May 5;18:10. doi: 10.1186/s41479-026-00199-x (PMC13141523; doi:10.1186/s41479-026-00199-x)

**Figure S1.** Flowchart of the study population

Excluding 182,807 patients with coronary heart diseases before the index date, including a period of five years before the study period (2002–2006).

Individuals aged 35–75 years with a registered healthcare contact in Sweden 2007–2018.

n = 4,843,859

**Study population**

n = 4,580,606

**Study population**

n = 4,661,052

Pneumonia

n = 1,480,721

No pneumonia

n = 3,099,885

Diabetes mellitus

n = 104,598

Diabetes mellitus

n = 243,426

Pneumonia

n = 1,512,693

No pneumonia

n = 3,148,359

Coronary heart disease

n = 94,087

Coronary heart disease

n = 201,505

Excluding 263,253 patients with diabetes mellitus before the index date, including a period of five years before the study period (2002–2006).

**Figure S2.** Kaplan-Meier survival estimates for diabetes mellitus in patients with and without a preceding pneumonia diagnosis


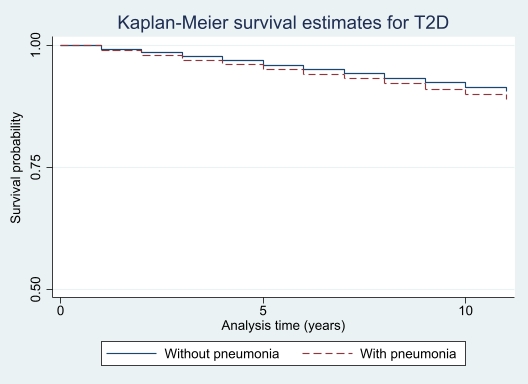


**Figure S3.** Kaplan-Meier survival estimates for coronary heart disease in patients with and without a preceding pneumonia diagnosis


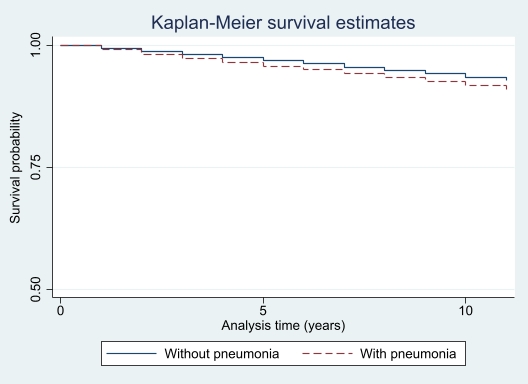

Supplement: Supplementary file 2 — Supplementary Material 2 [file 41479_2026_199_MOESM2_ESM.docx]
